# Diseases of the Dental Pulp and Their Treatment

**Published:** 1856-01

**Authors:** Chapin A. Harris


### AMERICAN JOURNAL

OF

### DENTAL SCIENCE.

**Vol. VI. NEW SERIES—JANUARY, 1856. No. 1.**

### ORIGINAL COMMUNICATIONS.

### ARTICLE I.

*Diseases of the Dental Pulp and their Treatment.* By CHAPIN A. HARRIS, M. D., D. D. S.

[Continued from page 531, of Vol. 4, New Series.]

AT the request of the Associated Alumni of American Dental Colleges, the writer prepared and read, at their second annual meeting, a paper on the diseases of the dental pulp. This paper was published in the April and July Nos. vol. 4, New Series, of the Journal, but as only two of the pathological conditions of the organ, *irritation* and *inflammation,* were noticed, he intended to resume, at some future period, the consideration of the subject, but arduous professional and other duties have hitherto prevented him from doing so. At the conclusion of the paper, a few remarks were offered on chronic inflammation, and a brief notice of the method of procedure pursued by Drs. W. W. Codman, of Boston, and W. H. Dwinelle, of Cazenovia, N. Y., in the treatment of exposed dental pulp. A few additional remarks upon this part of the subject will now first claim the consideration of the writer.

When the pulp of a tooth becomes exposed, it is very liable, as stated in a preceding part of this paper, to become the seat of chronic inflammation, and when this happens, the exposed diseased surface pours out serous fluid, rendering the operation of filling, during the continuance of the morbid action, impracticable, as the accumulation of fluid between the filling and the pulp would become an additional source of irritation and soon give rise to a more active form of inflammation. If, therefore, we would preserve the vitality of the tooth, our attention must first be directed to the restoration of the pulp to a healthy condition. This accomplished, the cavity may be filled with a fair prospect of success, but in doing this, care must be taken not to wound the exposed delicate, and, under all circumstances, highly sensitive organ.

Creosote and the essential oils of cloves, cinnamon and cajuput, from their pungent and stimulating properties, have long been held in high repute as odontalgic remedies, but the writer has seldom derived much benefit from them in the treatment of chronic inflammation of the pulp. The application of them, it is true, is often followed by an immediate and complete subsidence of the pain occasioned as a consequence of it, but the diseased action is seldom removed by their use. Dr. Dwinelle recommends, in cases of this kind, the direct application of tannate of lead, and, in two or three cases, the writer has used it with complete success. Still, he has not employed it in a sufficient number of cases, to enable him to speak very decidedly with regard to its merits, having failed oftener than he has succeeded, in attaining the object proposed by its use. A saturated solution of tannic acid in spirits of wine with a sufficient quantity of gum benzoin dissolved in it, to make it of the consistence of thick mucilage, applied on a little raw cotton, has been attended with more satisfactory results in the practice of the writer than tannate of lead.* A two-fold benefit is often obtained by the use of this preparation; for, while the stimulating properties of the

* The use of this preparation of tannic acid was recommended to the writer a few years since by Dr. F. H. Badger, of Nashville, Tenn., but not as a remedy for chronic inflammation of the pulp of a tooth.

alcohol have a tendency to allay pain, the astringent effect exerted by the tannin promotes resolution of the exposed inflamed surface of the pulp. Of the several cases successfully treated with it, it may not be amiss to give a brief history of one or two.

Mr. E., a young gentleman about twenty-one years of age, of a sanguino-nervous temperament, applied to the writer in the early part of the summer of 1854, for professional advice in relation to his left second upper bicuspis. It was decayed in its posterior approximal surface, and some eight or ten months before, the decomposed portion had been removed with a view to having it filled, but as the pulp was found to be considerably exposed, the operation was deemed impracticable. The first molar being in a carious condition and the anterior half broken off, the cavity in the bicuspid was completely exposed to view. As the young gentleman refused to submit to the loss of the tooth, it had been permitted to remain in this condition, though it had subsequently, several times ached, and occasioned considerable annoyance. The pain, however, was usually readily allayed by the application of stimulants, and hence the organ had been permitted to remain in his mouth.

After removing some foreign matter which had accumulated in the cavity of the tooth, the exposed part of the pulp could be readily seen. It protruded slightly into the external cavity, presenting a thickened and inflamed appearance. Regarding the constitutional temperament of the patient as unfavorable for the retention of a tooth after the destruction of its vitality, the writer determined to attempt to subdue the inflammation, and fill the tooth over the exposed pulp. With a view to which, he filled the cavity with raw cotton, saturated with the solution of tannin and benzoin, renewing the application every eight or ten days. At the expiration of four or five weeks, the exposed portion of the pulp assumed a healthy appearance, and had receded back into the central cavity. The tooth was now filled with Hill's stopping, leaving a small vacant space between the pulp and bottom of the filling, intending ultimately to replace it with gold. In three or four days Mr. E. returned, complaining of pain in the tooth. Supposing it to be occasioned by pressure of accumulated fluid at the bottom of the cavity, the Hill's stopping was removed and the solution applied as before. This treatment was continued several weeks longer, when the tooth was again filled with the preparation used in the first instance, and the patient requested to return immediately, if pain should a second time be experienced, and if it gave him no inconvenience, at the expiration of five or six months. As nearly as the writer can recollect, about nine months elapsed before he again saw him, and as the tooth had given no further trouble, and the filling still remained perfect, he was directed to leave it six or eight months longer, when the writer proposed to remove it and fill the tooth properly with gold. His object in delaying the operation so long, was to give ample time for the formation of a solid protective covering over the exposed part of the pulp. The patient not having yet returned for the replacement of the temporary with a permanent filling, the treatment may be regarded as successful.

Miss W., a young lady about twenty years of age, of a sanguino-bilious temperament and good constitutional health, applied to the writer in the winter of 1854, for his professional services. Several of her teeth were slightly affected with caries, and in one, the first right inferior molar, the disease had penetrated from the anterior approximal surface to the pulp-cavity. This tooth had ached several times, and though the exposed part of the pulp had become the seat of chronic inflammation and was aching at the time, as she was unwilling to lose the tooth, he applied to it, after having first removed all the decomposed dentine, the solution of tannin and benzoin, in the manner as described in the foregoing case, but as the pain was increased by the application, it was removed, and creosote substituted, closing the orifice of the cavity with yellow wax. The pain now subsided almost immediately, and there was no return of it the following day, the cotton on which the creosote was applied was removed, and the cavity filled with another piece saturated with the solution of tannin. This application was renewed every few days for three weeks. The exposed pulp having by this time assumed a healthy appearance, a gold filling was at once introduced. The tooth, when the writer last saw the young lady, more than twelve months after the operation had been performed, had given her no trouble and was as useful as any of her other teeth.

In the use of tannin in the form here described, there is no necessity for closing the orifice of the cavity with wax to exclude the secretions of the mouth, as the fibres of the cotton, as the alcohol evaporates or is diluted with the saliva, become agglutinated to each other by the benzoin. It answers the same purpose as gutta percha in chloroform.

In describing the successful results which have attended the use of the preparation last noticed in the treatment of chronic inflammation of the dental pulp, it is proper to state, that it has not proved thus efficacious in all the cases in which it has been employed by the writer. It has failed to accomplish the object proposed in a majority of the cases in which he has used it. Still, the beneficial effects which have resulted from its employment, entitle it to a place among the remedial agents resorted to in the treatment of the affection under consideration.

But the first step to be taken in the treatment of chronic inflammation of the pulp, as intimated in a preceding part of this paper, is to free the cavity in the tooth of all accumulations of extraneous matter and decomposed portions which can act as irritants, or aggravate the already existing disease. This done, it may be carefully washed out with tepid water and the remedial agent applied as previously directed. The success of the treatment too, will depend greatly upon the state of the general constitutional health of the patient at the time. An irritable condition of the system or derangement of the digestive organs will, in the majority of cases, render unavailing the most judicious and skillful treatment that can be adopted.

Dr. Koecker regards the preservation of a tooth as practicable, so long as the pulp is not actually in a state of suppuration or deprived of vitality, and he expresses the belief that as many as five out of every six cases, in which caries has penetrated to the lining membrane, may be preserved alive, and it is evident from the description which he gives of some of the cases he treated, that the pulp was in a state of chronic inflammation at the time. The method of procedure which he recommends consists in first cauterizing the exposed surface with a red hot wire, using the precaution not to touch any part of the surrounding dentinal walls or to wound the lining membrane; the exposed pulp is then covered with a plate of thin leaf lead, with the edges resting upon the surrounding solid parts, and the cavity filled with gold in the usual manner. In the commencement of his practice, Dr. K. states that he was in the habit of using tin foil, but as he was seldom successful, he was induced to substitute lead, believing that the last named metal exerted a "cooling and anti-inflammatory effect upon the irritated nerve of the tooth." Having never adopted this treatment in chronic inflammation of the pulp, the writer is unable to speak from experience, of its relative success as compared with the method of procedure which he has pursued in similar cases.

A solution of sulphate of zinc in rose water, in the proportion of from six to eight grains of the former to an ounce of the latter with thirty-five or forty drops of laudanum, may sometimes be employed with advantage when the tooth is free from pain. It acts as a cooling astringent, producing a very pleasant effect. The cavity in the tooth, however, should be first properly prepared. It is applied like most other remedial agents on a little raw cotton, sealing up the orifice with wax or mastic to exclude the secretions of the mouth. It causes for a few minutes, when first applied, a slight burning sensation, but this very soon subsides, leaving the tooth entirely free from pain. It should be applied once a day until the desired effect is produced, and in closing the orifice of the cavity care must be taken not to press upon the exposed pulp, as the irritation which would be thus produced would counteract the beneficial effects of the preparation. This may always be prevented by placing a cap of tin foil over the dossil of cotton before the orifice is closed.

*Ulceration.*—It is seldom that the pulp of a tooth can be restored to health after it has become the seat of ulceration.

The difficulty of applying to it suitable remedial agents and protecting it from the action of irritants, such as particles of foreign matter and the secretions of the mouth, increases the difficulty with which the practitioner has to contend, and very often renders unsuccessful, remedies, which, under other circumstances, might produce the desired effect. Still, ulceration, even of this most exquisitely sensitive tissue, is sometimes cured, and when the preservation of the tooth is called for by some peculiar or urgent necessity, every resource of the dentist should be called into requisition for the accomplishment of the object.

The treatment usually adopted in cases of this kind consists in the application of escharotics to the ulcerated surface of the pulp. Nitrate of silver has sometimes been successfully employed. It is used in a diluted state, in the proportion of from six to ten grains to an ounce of distilled water. It should be applied every day until the ulcer is healed, and the cavity in the tooth, after each application carefully closed to exclude all extraneous matter. The tooth, too, previously to commencing the use of it should be freed from all decomposed portions of dentine. Chloride of zinc, has also been used with advantage, but it is less efficient than nitrate of silver.

The application of the actual cautery, as recommended by Dr. Koecker, may, perhaps, after all, be better calculated to bring about a favorable result than any other remedial agent, but it must be very carefully and skillfully applied, to prevent wounding the pulp or touching any other part than the ulcer itself. The dentist having determined to apply it, should first prepare the cavity in the tooth by the removal of all decomposed portions of dentine, and being now provided with a lighted tallow candle, he holds in the flame of it the point of an iron wire until it attains a red heat, then applies it, for an instant only, directly to the ulcer. If this is followed by any bleeding, it is touched a second, and if necessary a third time, in quick succession. This done, the cavity in the tooth is made perfectly dry, a small piece of leaf lead placed over the bottom and the filling immediately introduced. This method of treatment, according to Dr. Koecker, proved eminently successful in his hands. The writer, however, has never tried it. Indeed, until within the last three or four years, he doubted the practicability of preserving the vitality of a tooth after ulceration of the pulp had taken place, but having been convinced that it can sometimes be done, he no longer doubts the truth of Dr. Koecker's statement. But the use of the actual cautery can only be resorted to in those cases where the diseased surface of the pulp is easy of access.

Should the foregoing methods of treatment fail to restore the pulp to a healthy condition, the vitality of it may be destroyed, either by the application of arsenious acid, or direct extirpation, if the preservation of the tooth be deemed a matter of sufficient importance, and as this often becomes necessary, a brief description of the manner of doing it, and the subsequent treatment may now very properly be given.

*Destroying and Extirpating the Pulp, and Filling the Root.* Immediate extirpation of the pulp is attended with more pain than the destruction of its vitality with arsenic, and is seldom resorted to in a tooth having more than one root. It is effected by thrusting a very delicate untempered spear pointed steel instrument directly into the nerve cavity to the extremity of the root, then it is severed by a few rotary motions, and if not brought away in the withdrawal of the instrument, may be removed with another having several sharp barbs cut upon it near the point. Some dentists think this method preferable to the other, and better calculated to secure the subsequent preservation of the tooth, but others who have practiced both, deny that the former possesses any advantages whatever over the latter. It is supposed by some that the effects of the arsenious acid extend to the peridental membrane, impairing, if not destroying, its vitality and rendering the tooth obnoxious to the parts within which it is implanted. But this effect can only be produced by a larger quantity, and permitting it to remain longer in the tooth than is necessary to accomplish the object proposed by its employment.

When the pulp is destroyed by direct extirpation, the tooth may be filled immediately or as soon as the oozing of blood from the mouths of the wounded vessels at the extremity of the root ceases; the effusion of lymph which takes place during the curative process is seldom sufficient to cause irritation.

In destroying the pulp with arsenious acid it is not necessary to use a very large quantity; the thirtieth part of a grain is amply sufficient, combined with an equal quantity of sulphate of morphia. For convenience, have a grain of each thoroughly incorporated, by grinding in a small mortar, then divided into thirty equal parts and each put up in a small piece of paper and kept ready for use. One of these is applied on a dossil of raw cotton, moistened with creosote, or oil of cloves or cajuput and applied directly to the exposed part of the pulp. Over this, a small cap of lead or tin is placed and the cavity in the tooth filled with wax or Hill's stopping, to exclude the buccal secretions and prevent the arsenic from escaping into the mouth. The advantage derived from placing a cap of lead or tin over the arsenic is the prevention of pressure on the pulp in filling the cavity with wax, and the consequent freedom from pain during the destruction of its vitality. When this is done, the tooth rarely aches during the action of the arsenic. With proper care the cavity may, in nineteen cases out of every twenty, be filled with wax or any other plastic substance, when the application of a protective covering to the pulp is omitted, but by using such precaution the liability of it is prevented, and the trouble of applying it is so trifling that it should always be done, at least, by the inexperienced practitioner.

At the expiration of from six to ten hours, the arsenic may be removed and the cavity washed with tepid water. This done, the opening into the central chamber of the tooth is enlarged by cutting away the solid dentine, with instruments properly adapted to the purpose, and when practicable, without wounding the dead pulp, which may, afterwards, in many cases, be brought away almost entire. Every particle of it, however, should be removed to the very extremity of the root or roots, if the tooth have more than one. With a view to which, untempered steel instruments with barbed points, and sufficiently small to traverse the canal in the root, is introduced and withdrawn several times or until the operator is absolutely certain that no portion of the disorganized pulp remains. This part of the operation is sometimes exceedingly difficult, requiring the patient exercise of no little ingenuity and skill, especially when the tooth is a bicuspid or molar, having two or more roots, and the opening through the crown in the posterior approximal surface. Indeed, it cannot always be done without first filing away one-fourth or one-third of the crown, and increasing the size of the external opening.

In a lower molar the anterior and posterior walls of the canal in the roots, almost meet in the centre, leaving a very delicate opening or canal on each side, from both of which, the elongated pulp is to be carefully removed. This, also, is sometimes the case in a bicuspid, so that instruments not larger than a small bristle are often required. When the root is bent or curved, the difficulty of traversing it to its extremity, is, of course, very greatly increased. The canal in the buccal root of an upper molar is not unfrequently so small that it cannot be penetrated through its entire length, even by the most delicate instrument that can be made. In this case the dentist must be content with the removal of as much of the hair-like elongation of the pulp passing through it as he can bring away, and, fortunately, any portion which may afterwards remain is too small to be productive of serious injury.

The arsenic should never be permitted to remain in the tooth longer than from ten to sixteen hours, and in most cases from six to seven will suffice for the complete destruction of the vitality of the pulp, and when placed directly upon it, a second application rarely if ever becomes necessary.

There is almost always, after the removal of the pulp, a slight oozing of blood from the mouths of the severed vessels at the extremity of the root. This sometimes continues for two or three days. It is, therefore, necessary to delay the subsequent operation of filling, as has been already intimated, until it ceases. The blood which at first escapes does not coagulate, but as the vessels at the apex of the root recover from the effects of the arsenic and acquire a healthy tone, their extremities soon cicatrize.

The oozing of blood having ceased, the pulp cavity may be syringed out with water and then dried with prepared raw cotton, but the most convenient way of introducing this into the root,* is to wind a small quantity on a probe small enough to penetrate readily to its apex, and made rough to prevent the cotton slipping off when the instrument is withdrawn. This done, the operation of filling may be commenced, but in introducing the gold, different practitioners have adopted different methods of procedure. Some adopt the method proposed by Dr. Maynard, of introducing very thick foil, say No. 15, 20 or 30, cut in strips not wider than the diameter of the smallest part of the canal in the root; others use it rolled into small cylinders as recommended by Dr. F. H. Badger, and others again prefer to introduce it in the form of small pellets. But whatever be the form in which it is used, it is necessary to consolidate it sufficiently to render it impermeable to fluids. When narrow strips are used, the end of one is placed upon the point of an untempered steel filling instrument, of the proper size, and carefully carried to the apex of the root. The instrument is now withdrawn a short distance and again returned, carrying with it another fold, and this is repeated until the entire strip has been introduced, compacting each successive fold as firmly as the instrument will permit. Thus, strip after strip is introduced until the canal is filled. If the tooth has more than one root, the filling of the others may be deferred until a future sitting of the patient. A sitting may be alloted to the filling of each root, or all may be filled at one time. But in filling the roots and pulp cavity of a molar, two or three sittings are sometimes necessary, and having completed these several parts of the operation, the cavity in the crown is filled in the usual manner. The most skillful manipulation is required in every part of the operation, especially when the tooth has more than one root.

*Cotton used for drying cavities in teeth preparatory to filling, should have the oil removed from it by boiling a few minutes in a tolerably strong solution of carbonate of soda, or some other alkali, to make it absorb moisture freely.

When the gold is used in the form of a cylindrical cone, a piece is cut from the leaf, of a triangular shape, and rolled as compactly as possible, using the precaution not to make it too large to prevent it from being easily introduced to the extremity of the root, nor the point so sharp and small as to admit of its being carried above the apex of the fang, as in that case, it will act as an irritant to the periosteal tissues of the alveolus. When there is danger of this, the point should be clipt off with a pair of scissors before it is introduced into the root. The gold being thus prepared, an untempered stilet is introduced to the point intended to be reached by the filling, and the length of the canal indicated by bending the instrument at the commencement or mouth of the canal. The length is then marked on the cylinder of gold. This done, it is carried up to the designated point, then an untempered steel instrument, having a point shaped like the cylinder of gold, but a little smaller, is forced up between it and the root, compressing the former as much as the strength of the instrument will permit. Having proceeded thus far, another cylinder of gold is introduced, and this process is repeated until the cavity is compactly filled. The root or roots, if the tooth has more than one, being filled, the operation is completed by filling the pulp and crown cavities in the usual manner.

The third and last method to be noticed, consists in cutting the gold into small square pieces varying in size from a twelfth to a sixth or fourth part of an inch, depending on the thickness of the leaf and the size of the canal to be filled. These are carried up to their place in the root, one at a time, beginning with the smallest, on the point of a suitable instrument. Each one is compressed as much as the strength of the instrument will permit, as it is introduced.

The relative merits of these several methods of procedure depend very greatly upon the perfection in which each is practiced. The root of a tooth may be very compactly filled by either, but to do it, requires, as already intimated, much time, and the nicest skill in the management and working of the gold, especially when the canal is very small and difficult of access.

The operation is less likely to be successful when performed on the tooth of a person previously to the sixteenth or eighteenth year of age than at a later period, and for the reason that the canal in the root is, up to this time of life, much larger than after eighteenth or twentieth year of age, especially the opening at the extremity of the root. Hence, the root itself, after the destruction of the pulp, is more liable to act as an irritant, and there is greater danger of forcing the gold through it into the socket at its apex, no matter what may be the method in which it is used. An accident of this kind will defeat the success of the operation and render the extraction of the tooth necessary. To prevent which, when the canal is large, the length of it should always be ascertained in the manner as before directed, previously to introducing the gold; by using this precaution, it may be prevented.

In filling the root of a lower molar, when it becomes necessary to introduce two fillings in consequence of the approximation of the walls of the central part of the opening throughout its whole length, leaving only a very small canal on each side, the operation, very often, may be facilitated and rendered more perfect, by enlarging them with a watch-maker's broach. The same may also be done with the bicuspids under similar circumstances, and sometimes with the buccal roots of the upper molars. Indeed, the canal of the root of any tooth, may be enlarged in this way when necessary and easy of access.

The foregoing brief directions, intended only for the inexperienced practitioner, will, the writer trusts, be found sufficient to guide him in the performance of the operation, but cases will occasionally come up in practice where the ingenuity of the operator alone will have to determine the method of procedure most proper to be pursued.

*Fungous Growth.*—There is sometimes developed from the pulp of a tooth, after it has become exposed, and been in a state of chronic inflammation or ulceration for a greater or less length of time, a morbid growth, which assumes the form of a small vascular tumor of about the size of a duck shot or elderberry, and as sensitive to the touch as the pulp when in a healthy state. It rarely grows very rapidly, and never attains a very large size. It always proceeds from the exposed parts of the pulp and is preceded, usually, for several weeks by chronic inflammation or ulceration. The proper remedial indication consists, in the majority of cases, in the extraction of the tooth, though the fungous growth, it is probable, when wholly confined to the pulp, may sometimes be repressed by the application of escharotics or the actual cautery. The disease, however, is usually regarded as incurable.

It often happens that the central cavity of a tooth, after it has become exposed by the decay of the crown, is occupied by a morbid growth, which has originated from the periosteum of the root or gum, the caries having extended through the neck of the organ. Through this opening the fungous growth finds its way into the tooth, filling in a short time both the central and crown cavities. Tumors of this kind are frequently mistaken for a morbid growth of the pulp. They usually grow very rapidly, and sometimes attain a very large size. They are also very vascular and bleed freely when wounded. The writer has frequently met with tumors of this sort which had their origin in the alveolo-dental periosteum at the extremity of the root, the morbid growth having made its way through the enlarged canal into the crown. It is scarcely necessary to say, that fungous productions of this character can only be cured by the extraction of the tooth, inasmuch as they are always soon reproduced after removal. The teeth, too, in which they are met with are usually so much decayed as to preclude the possibility of their preservation.

*Spontaneous Disorganization.*—This affection, as the writer has stated in another place, seems to have been entirely overlooked by writers on dental pathology, and although it is one which rarely occurs, examples of it are met with sufficiently often to entitle it to a place among the diseases of the teeth. The first case to which his attention was particularly directed, occurred in 1836. Since which time, eight or ten other cases have fallen under his observation. In each, the disorganizing process was carried on so insidiously that neither structural alteration or the existence of diseased action of any kind was suspected, until the teeth assumed a dull bluish brown appearance. In neither case, so far as could be ascertained, was there the slightest indication of inflammatory action. The death was seemingly the result of the suspension of the nutritive function occasioned by the want of sufficient vital energy to carry it on.

In all the cases which the writer has seen, the sockets of the teeth were, apparently, in a healthy condition, the margins of the gums thin and regularly scolloped, without any indications of structural alteration, except that they had a dingy grayish-purple appearance, instead of a pale rose color, the aspect they exhibit around living teeth in good constitutions. The disorganized pulp does not, as in those cases where it has perished from inflammation or from the application of escharotics, seem to exert any morbid effect upon the parts at the extremity of the root. This, as he has elsewhere stated may be owing, in part, to diminished excitability in the alveolo-dental periosteum, and to the inoxious character of the disorganized matter. The death of the pulp in all the cases which have fallen under his observation occurred before the twentieth year of age. It rarely takes place in a single tooth, though examples are occasionally met with, but in the majority of cases it occurs simultaneously in corresponding teeth, the pulps of two or four usually perish at about the same time. In the first case which came under his observation, six had perished. The incisors, however, appear to be more particularly subject to it than the molars and bicuspids, and it occurs as frequently in sound as in decayed teeth.

The death of the pulp not being the result of inflammation, must be dependent upon constitutional rather than local causes, upon some impairment of the function of sanguinification or an exceedingly serous condition of the blood.

The subject of the first case seen by the writer was a young lady about eighteen or twenty years of age, of a chlorotic habit and having a slightly bloated aspect of countenance. The discoloration of the teeth had existed several years and was confined to the four upper and two central lower incisors. The parents of the young lady supposed it was occasioned by caries in the approximal surfaces, and it was for the removal of this that he was consulted, but the teeth, upon examination were, to their astonishment, found to be perfectly sound. Not the slightest indication of structural alteration in either the enamel or dentine could be detected. The cause of the discoloration was now ascertained to be the result of the disorganization of the pulps, and the removal of these seemed to constitute the only and proper remedial indication. With a view to which, a perforation was made in each of the upper incisors, from the palatine side to the central chamber, large enough to permit the complete removal of every thing contained in them. A drop or two of dark brown matter of about the consistence of thick cream, and almost wholly without odor, immediately escaped. The walls of these cavities were slightly discolored, but on being scraped they became as white, though not as translucent as healthy dentine. The natural color of the teeth having been very nearly restored, the pulp-cavities and roots were filled in the manner as already described. The two lower incisors not being so conspicuous were permitted to remain as they were.

It rarely happens that a tooth which has lost its vitality by the spontaneous destruction of the pulp, gives rise to the formation of alveolar abscess. The writer does not recollect of having met with a single case in which this has happened.

*Ossification.*—With regard to this affection the writer can only repeat what he has elsewhere stated upon the subject.* Ossification of the pulp of a tooth is a means employed by nature to prevent the exposure of this most exquisitely sensitive tissue. It is true, examples of it are occasionally met with in teeth which have not suffered any loss of substance, either from mechanical or spontaneous abrasion or from structural alteration of either the enamel or dentine. The occurrence, whatever may be the circumstances under which it takes place, is unquestionably the result of an established law of the economy, dependent, no doubt, upon moderate irritation and increase

* See Principles and Practice of Dental Surgery.

of vascular action. The deposition of earthy salts having commenced, it usually goes on until every part of the pulp is converted into a substance analogous to, if not identical with, cementum. Thus, it would seem, that when the pulp of a tooth becomes the seat of a sufficient amount of irritation, ossification follows as a necessary consequence, but if the irritation be succeeded by active inflammation, a different result takes place, namely, suppuration.

The irritation necessary to the ossification of the pulp of a tooth sometimes arises from constitutional causes, but in the majority of cases, it results from the action of local irritants, and most frequently, from impressions of heat and cold, communicated through the conducting medium of a metallic filling or a thin layer of dentine.

During the ossification, a sensation is occasionally experienced in the tooth somewhat similar, though altogether less in degree, to that which attends the knitting of the fractured extremities of a broken bone. A numb, slightly twinging pain, barely perceptible, is felt passing through the tooth several times a day, but lasting only a second or two at a time. It is scarcely sufficient to occasion annoyance, or to attract any thing more than momentary attention.

With the ossification of the pulp, the crown and inner walls of the root lose their vitality, but the appearance of the tooth is not, as in the case of necrosis arising from disorganization of the pulp, materially effected. The central cavity being filled with semi-translucent bone, or osteo-dentine, the crown retains its natural color. The discoloration and opacity attending necrosis, arising from other causes, results, partly from the presence of putrid matter in the pulp-cavity, and partly from its absorption by the surrounding dentinal walls.

There are several beautiful examples of ossification of the pulps of teeth in the Museum of the Baltimore College of Dental Surgery, and the writer has some eight or ten in his own private cabinet.

